# Slit Guidance Ligand 3 (SLIT3) Loaded in Hydrogel Microparticles Enhances the Tendon-Bone Healing through Promotion of Type-H Vessel Formation: An Experimental Study in Mice

**DOI:** 10.3390/ijms241713638

**Published:** 2023-09-04

**Authors:** Jianting Li, Guanfu Wu, Changhao Xu, Zhining Cai, Jiali Ji, Ziyi Yu, Jing Zhang, Jiali Wang

**Affiliations:** 1 School of Biomedical Engineering, Sun Yat-sen University Shenzhen Campus, Shenzhen 518107, China; lijt58@mail2.sysu.edu.cn (J.L.);; 2 State Key Laboratory of Materials-Oriented Chemical Engineering, College of Chemical Engineering, Nanjing Tech University, 30 Puzhu South Road, Nanjing 211816, China

**Keywords:** type H vessels, tendon–bone healing, slit3, HMP

## Abstract

Poor tendon–bone interface (TBI) integration is one of the major causes contributing to unsatisfactory healing quality in patients after anterior cruciate ligament (ACL) reconstruction. Type H vessels have been recently found to closely modulate bone formation via regulation of the osteo-angiogenic crosstalk, so the strategies favoring type H vessel formation may be promising therapeutic approaches for improved graft osteointegration. In this study, we reported for the first time the treatment outcome of slit guidance ligand 3 (slit3), a novel proangiogenic factor favoring type H vessel formation, in TBI healing in mice with ACL reconstruction. The mice (n = 87) were divided into three groups for various treatments: hydrogel microparticles (HMP, control group), slit3@HMP, and slit3 neutralizing antibody@HMP (slit3-AB@HMP). Histological analysis, gait performance, radiographic measurement, and biomechanical testing were performed to assess the TBI healing quality. Increased bony ingrowth and reduced fibrous scar tissue was formed at the TBI in the slit3@HMP group when compared to the HMP group. Meanwhile, the slit3-AB@HMP inhibited the osseous ingrowth and increased fibrous scar tissue formation relative to the HMP group. Compared to the HMP group, the slit3@HMP favored type H vessel formation at the TBI while the slit3-AB@HMP impeded it. According to micro-CT assessment, compared to the HMP group, the slit3@HMP significantly increased the peri-tunnel bone mass while the slit3-AB@HMP significantly reduced the peri-tunnel bone mass. The mice in the slit3@HMP group showed the best gait performance in terms of stance time, stride length, paw print area, and stance pressure. Dynamic laxity measurement and tensile testing showed the slit3@HMP group exhibited significantly reduced laxity displacement and improved failure load and stiffness relative to the other two groups. Collectively, the injection of slit3 could be used to enhance tendon–bone integration, which may be ascribed to modulation of angiogenesis–osteogenesis crosstalk coupled by type H vessels.

## 1. Introduction

Anterior cruciate ligament (ACL) injuries are a common sports-related injury affecting over 100,000 patients each year, and reconstruction is often necessary to restore knee stability due to the poor healing capacity of the ACL [1,2]. The estimated failure rate of reconstructed tendon grafts is approximate 10–15% due to poor graft healing at the tendon–bone enthesis, thereby leading to requirement of a second revision surgery [3]. The enhancement of graft osteointegration into bone tunnels is a promising therapeutic strategy to improve the long-term healing quality in patients after reconstruction [4]. Increasing evidence provides proof of the basic principle that promotion of the neovascularization at the interface between the tendon graft and bone tunnels favors the support of sufficient oxygen and nutrients to the cells [5,6], thereby contributing to more bony ingrowth. However, as the proangiogenic factors also induce inflammatory responses, they may exert dual-phase effects on the tendon–bone healing.

For instance, blocking vascular endothelial growth factor (VEGF), one commonly used angiogenic factor, significantly reduced angiogenesis, graft maturation and biomechanical strength following ACL reconstruction [6], and injection of intra-tunnel VEGF accelerated the graft healing process [7]. Of note, as one pro-inflammatory cytokine, VEGF may induce osteoclastogenesis of the osteoclast precursors, which could thereby promote the secretion of PDGF-BB favoring type H (CD31^hi^EMCN^hi^) vessel formation [8]. However, the over-expression of VEGF impeded improvements in tensile strength [6]. Similarly, exogenous VEGF delivery in sheep with ACL reconstruction also increased newly formed vessels while inducing negative mechanical effects [9]. The biphasic model of VEGF may be ascribed to its pro-angiogenic and pro-inflammatory effects, thus resulting in excessive infiltration of fibroblasts in the long-term intervention [10].

Recently, it has been reported that the type H vessel favors bone regeneration through modulation of angiogenesis–osteogenesis coupling effects [11,12]. Type H blood vessels are mainly distributed in the metaphysis, periosteum, and endosteum [13]. In ACL reconstruction, the bone tunnel will pass through the metaphysis region with abundant type H vessels, indicating that CD31^hi^Emcn^hi^ endothelium may be involved in the angiogenesis during tendon–bone healing. Slit guidance ligand 3 (slit3), derived from osteoblasts or osteoclasts [14], is coupled to anabolic bone formation via activation of ERK mitogen-activated protein kinase and Hippo signaling through Robo family receptors, thereby enhancing type H vessel formation [15]. Although platelet-derived growth factor BB (PDGF-BB), mainly secreted by mononuclear preosteoclasts, can also induce type H vessel formation during bone remodeling [8], it constitutes as a key pro-inflammatory cytokine in concert with transforming growth factor beta (TGFβ) to induce fibrosis [16]. Of note, the extensive deposition of fibrous connective tissue initiated by macrophage activation impairs tendon–bone healing [17]. Therefore, the delivery of slit3 to bone tunnels may be a better therapeutic option beneficial for angiogenesis relative to the PDGF-BB administration. To our best knowledge, this is the first report on the strategy involving the modulation of a specific vessel subtype, the CD31^hi^EMCN^hi^-expressing type H vessel, for enhancement of the graft osteointegration. We raised the hypothesis that the delivery of slit3 could accelerate and promote the osseous ingrowth towards the TBI through increasing type H vessels at the graft enthesis. Accordingly, we encapsulated slit3 or its neutralizing antibody in the micro-hydrogels, for an extended release, prior to injection into the bone tunnels of mice with ACL reconstruction. The immunofluorescence staining, radiographic measurement, gait analysis, and biomechanical testing were applied to assess the treatment outcomes and verify if slit3 affects the graft healing via modulation of type H vessel formation.

## 2. Results

### 2.1. Characterization and Biocompatibility of HMP

**Figure 1:**
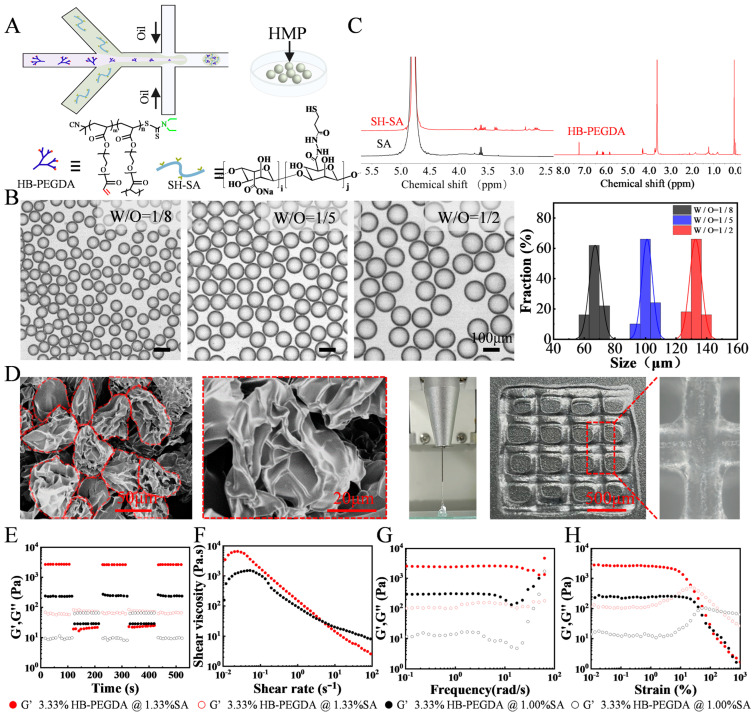
Preparation of the HMPs and their favorable rheological properties. (**A**) Schematic for the preparation of HMPs using a microfluidic device. (**B**) The HMPs with different particle sizes were attained by adjusting water-oil flow rate ratios. (**C**) ^1^H NMR (400 MHz) spectra of thiolated sodium alginate (SA-SH) in D_2_O and hyperbranched (polyethylene glycol) diacrylate (HB-PEGDA) in CDCl_3_. (**D**) The SEM images of the resulting HMPs. (**E**–**H**) Rheological characterizations of the HMPs.

We employed a microfluidic water-in-oil emulsion technique, depicted in Figure 1A, to divide a continuous pre-gel aqueous phase into consistent hydrogel microparticles. By generating HMP sequentially at the microscale using microfluidic droplet technology, instead of relying on conventional methods involving mechanical stirring and sonication, we achieved precise regulation over the formation conditions and the resulting material properties of the final HMP. During this experiment, a diverse array of HMPs with different particle sizes was attained by varying the water–oil flow rate ratios. The water flow rate remained constant at 6 μL/min, while the oil flow rate was adjusted to 48 μL/min (W/O = 1/8), 30 μL/min (W/O = 1/5), and 12 μL/min (W/O = 1/2), respectively. For each set of conditions, a total of 50 microgels were analyzed to determine their size distribution. (as illustrated in Figure 1B). The resulting HMPs consisted of a hydrogel network comprising sodium alginate functionalized with thiol groups (SH-SA), which incorporated slit3 or its neutralizing antibody (Figure 1C). Crosslinking of the microgels was accomplished through a gentle gelation process using vinyl-terminated hyperbranched (polyethylene glycol) diacrylate (HB-PEGDA), ensuring the preservation of protein bioactivity [18]. Moreover, in the pursuit of obtaining jammed granular hydrogels, a purification process was employed on the HMPs using an aqueous solution of cell culture media, followed by their concentration through centrifugation. This meticulous procedure aimed to ensure the removal of any impurities and enhance the uniformity of the resulting hydrogels. The effectiveness of this purification approach is evident from the scanning electron microscopy (SEM) images depicted in Figure 1D, which clearly illustrate the discrete and well-dispersed nature of the HMP building blocks within the granular hydrogels. Their rheological characteristics were also assessed. Figure 1E presents the results of the oscillatory amplitude sweep measurements, illustrating the changes in the dynamic storage modulus (G′) and loss modulus (G″) as a function of shear strain (γ). These tests were conducted within a range of strains encompassing both small and large amplitude deformations, to gain a comprehensive understanding of the linear viscoelastic (LVE) and non-LVE behaviors of the granular hydrogels. The consistent plateau values observed at low strains indicate predominantly solid-like elastic response within this strain range. As the deformation amplitudes increase, G′ decreases due to the collapse of the HMP jamming structure. Notably, when the SA concentration was raised from 1% (*w*/*v*) to 1.3 wt%, a three-fold increase in the storage modulus was observed, indicating the ability to easily adjust the mechanical strength of the resulting granular hydrogel by altering the pre-gel concentration (Figure 1F).

Subsequently, to examine the self-healing properties of the granular hydrogel, an oscillatory strain was applied, alternating between 1000% and 0.1% at a frequency of 1 Hz. Figure 1G demonstrates that under high magnitude strain (1000%), the granular hydrogel displays shear-thinning behavior, as indicated by G″ > G′, suggesting a sol state. Interestingly, when the strain is switched to a low magnitude (0.1%), the sol promptly transitions back to a gel state, indicating its self-healing capability. Furthermore, we conducted a continuous flow experiment to assess the instantaneous viscosity modulus at a controlled frequency of 1 Hz and a wide range of shear rates spanning from 0.01 to 100 s^−1^. As depicted in Figure 1H, the gene therapy gels exhibit favorable shear-thinning performance. This characteristic implies that the granular hydrogel becomes more viscous and easily flows under external shear forces, which is advantageous for in vivo injection. Once the external force is removed, the gel swiftly regains its elasticity, ensuring localized drug retention. The injectability of the granular hydrogels is illustrated in Figure 1D showcasing the ability to create customized 2D patterns using an extrusion 3D printer in conjunction with a 26-gauge syringe needle. Owing to the relatively weak physical interactions among the building blocks of the HMP, the printed lattice gradually collapses over time (as observed in the high-resolution image on the right side of Figure 1H). However, owing to the micron size of the HMP particles, they are capable of being retained locally after injection into the body.

**Figure 2:**
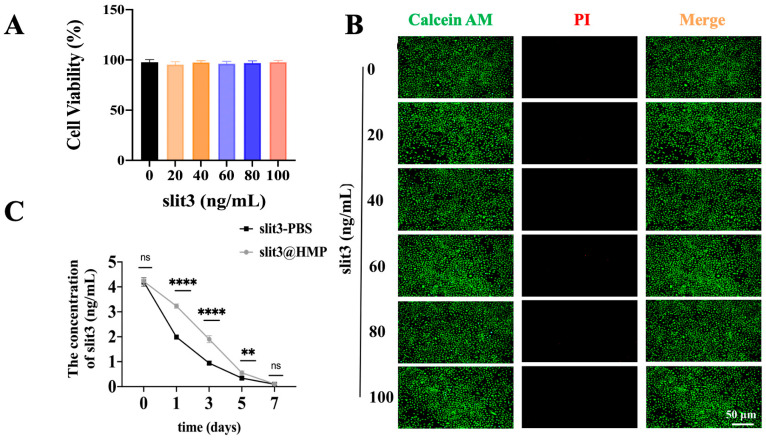
Biocompatibility assessment and stability examination of slit3 loaded in HMPs. (**A**,**B**) Cytocompatibility assessment of the HMPs loading different doses of slit3 by using CCK-8 testing and Live/Dead staining assay. HUVEC was used for the assay. All the scale bars are 50 μm. (**C**) In vitro stability measurement of slit3 alone and slit3 loaded in HMPs after immersion into PBS at 4 °C. ns: not significant, ** *p* < 0.01, **** *p* < 0.0001. n = 5 individual biological replicates.

Of note, the HMPs loading different doses of slit3 exhibited excellent cytocompatibility even when the dose of slit3 in the medium reached 100 ng/mL (Figure 2A,B). Importantly, the HMPs effectively prolonged the stabilization of the exogenous slit3 (Figure 2C), indicating an extended in situ release of slit3 loaded in HMPs relative to slit3 alone at the TBI microenvironment.

### 2.2. Histological Evidence of Graft–Bone Integration

Histological evaluation was performed with Hematoxylin and Eosin and Safranin O and Fast Green staining methods to assess the graft healing quality at 2, 4, and 6 weeks after surgery. During the whole observation period, there was no fibrocartilage formation at the TBI even along the longitudinal direction of bone tunnels.

**Figure 3:**
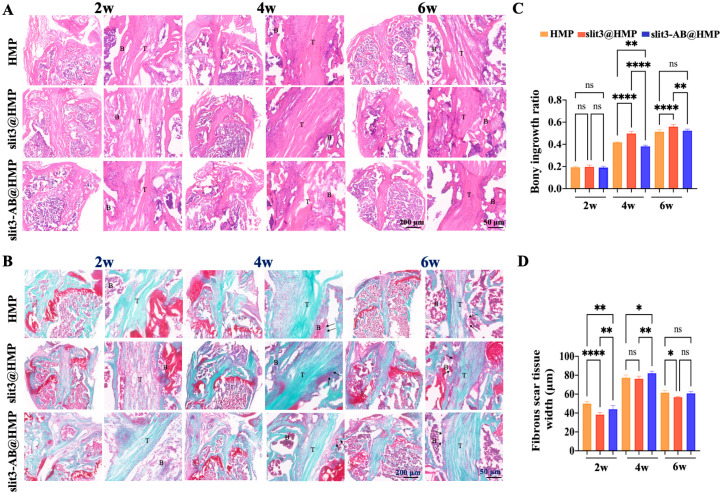
Histological analysis of tendon–bone interface in the HMP, slit3@HMP and slit3-AB@HMP group at 2, 4, and 6 weeks in mice that underwent ACL reconstruction. (**A**) Hematoxylin and eosin (HE) staining of the tibial tunnel interface. (**B**) Safranin O fast green (SOFG) staining of tibial bone tunnel interface. Scale bars, 200 μm and 50 μm. (**C**) Quantitative analysis of bony ingrowth ratio and (**D**) fibrous scar tissue width in the HMP, slit3@HMP, and slit3-AB@HMP groups. * *p* < 0.05, ** *p* < 0.01, **** *p* < 0.0001, ns: not significant. n = 5 individual biological replicates. T: tendon graft; B: bone. The arrows indicated the newly formed bone at the tendon-bone interface.

The bony ingrowth at the TBI and the fibrous scar tissue width were quantitatively measured for assessment of the graft integration quality. As shown in Figure 3A,B, the local injection of slit3 significantly increased the bony ingrowth towards the TBI relative to the HMP group at 4 weeks and 6 weeks. Importantly, the bony ingrowth towards the TBI constantly improved over the healing time in the slit3-treated group, thereby contributing to enhanced osteointegration. On the contrary, the intervention of slit3 neutralizing antibody (slit3-AB) significantly impaired the osseous ingrowth into the TBI relative to the HMP group at 4 weeks (Figure 3C). Interestingly, the fibrous scar tissue width at the TBI in all the three groups increased from 2 weeks to 4 weeks after surgery, then decreased at 6 weeks post-operation. Compared to the HMP group, the slit3 significantly inhibited fibrous scar tissue formation while the slit3-AB significantly increased the scar tissue width at the TBI at 2 weeks (Figure 3C).

### 2.3. Type H Vessels at the TBI

**Figure 4:**
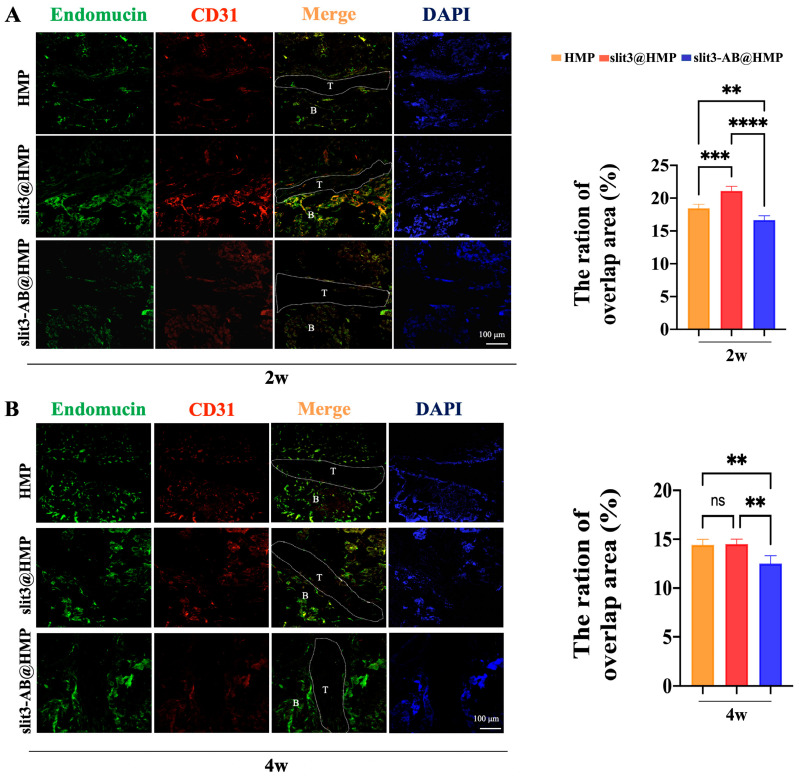
Representative immunostaining images and quantitative analysis of CD31^hi^Emcn^hi^ endothelium (type H vessels) at the TBI along the longitudinal direction in bone tunnels at the tibial side in mice at 2 weeks (**A**) and 4 weeks (**B**) post-surgery. T: tendon graft; B: bone, and white dots indicate the boundary of tendon–bone interface. Scale bar: 100 μm. ** *p* < 0.01, *** *p* < 0.001, **** *p* < 0.0001, ns: not significant, n = 3.

Although it has been widely reported that the type H vessels couple osteogenesis and angiogenesis in the bone microenvironment [13], it is still unclear whether there is type H vessel formation at the TBI during the graft healing after ACL reconstruction. Encouragingly, the CD31^hi^Emcn^hi^ skeletal endothelium was observed at the TBI in the HMP group at 2 weeks after surgery, indicating that the type H vessels may be also involved in the graft osteointegration into bone tunnels. Of note, as compared to the HMP group, the slit3 treatment dramatically increased CD31^hi^Emcn^hi^ endothelium at the TBI while the slit3-AB@HMP showed the opposite effects at 2 weeks after surgery (Figure 4A). Interestingly, the slit3 treatment did not promote more type H vessel formation at the TBI relative to the HMP group at 4 weeks post-surgery (Figure 4B). As expected, the signal assigned for CD31^hi^Emcn^hi^ positive endothelium at the TBI in the slit3-AB-treated group was still weaker relative to the other two groups.

### 2.4. The Peri-Tunnel Bone Tissue

**Figure 5:**
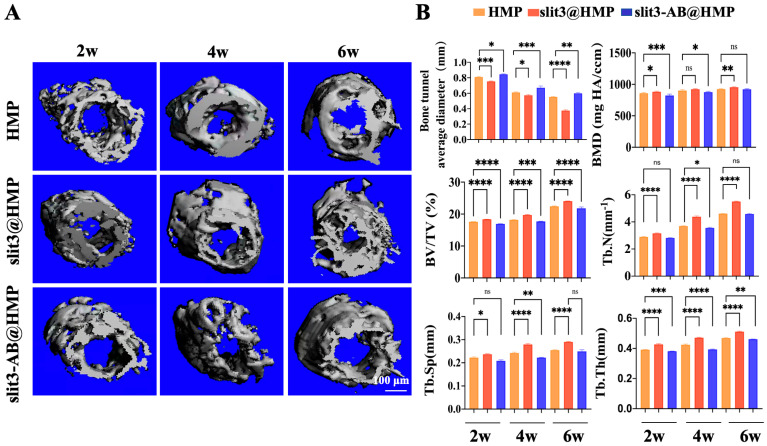
(**A**) Representative 3D reconstruction images of the tibial bone tunnel among HMP, slit3@HMP and slit3-AB@HMP group at 2, 4, and 6 weeks after surgery. Scale bar: 100 μm. (**B**) Comparison of the bone tunnel average diameter, BV/TV, BMD, Tb.N, Tb.Th, and Tb.Sp in new bone among the 3 groups (n = 5). * *p* < 0.05, ** *p* < 0.01, *** *p* < 0.001, **** *p* < 0.0001, ns: not significant.

The peri-tunnel bone mass is another important indicator for assessment of the graft healing. For instance, insufficient peri-tunnel bone mass implies unsatisfactory bony ingrowth towards the TBI and impaired bone microstructure under the bone tunnels, so the peri-tunnel bone tissue was scanned by micro-CT and then reconstructed for quantitative analysis of BMD, BV/TV, Tb.Sp, Tb.N, and Tb.Th (Figure 5A). In addition, the bone tunnel size was also measured. As shown in Figure 5B, the bone tunnel sizes in all the three groups at 2 weeks post-surgery were larger than those of the pre-drilled tunnels. However, the slit3 treatment significantly inhibited the enlargement of bone tunnels relative to the HMP or the slit3-AB treatment. In addition, the bone tunnel size gradually decreased over healing time. Importantly, the slit3 treatment significantly increased BMD, BV/TV, Tb.N, and Tb.Th while decreasing Tb.Sp of the peri-tunnel bone tissue relative to the HMP or the slit3-AB treatment. As expected, the slit3-AB treatment significantly decreased BMD, BV/TV, Tb.N, and Tb.Th while increasing Tb.Sp of the peri-tunnel bone tissue relative to the HMP treatment.

### 2.5. Gait Performance

**Figure 6:**
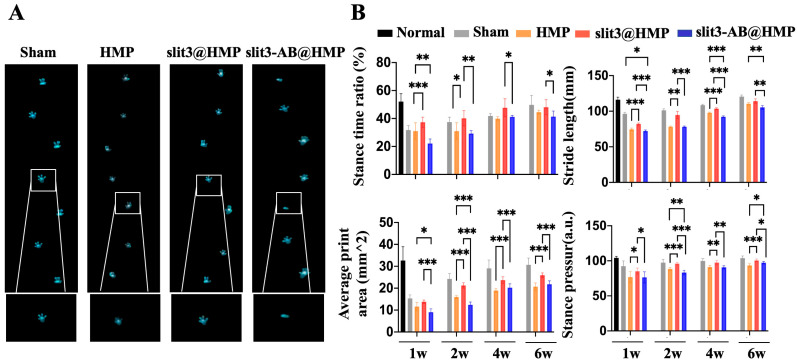
(**A**) Representative images showing the gait patterns of mice with or without ACL reconstruction in the Sham, HMP, slit3@HMP, and slit3-AB@HMP groups. (**B**) Quantification of key gait parameters for stance time ratio, stride length, average print area, and stance pressure in mice in the Sham, HMP, slit3@HMP, and slit3-AB@HMP groups at 1 week, 2 weeks, 4 weeks, and 6 weeks after surgery. The normal group referred to the mice without surgery. * *p* < 0.05, ** *p* < 0.01, *** *p* < 0.001. n = 6.

Gait analysis is a critical tool to test any gait changes and detect quantitative information about the animal’s behavior. Satisfactory graft osteointegration in mice that underwent ACL reconstruction may reduce knee joint laxity, thereby contributing to less alteration in gait performance relative to the mice without surgery. Therefore, we performed gait analysis in mice with different treatment strategies. As shown in Figure 6A, the paw-print signals were captured to measure stance the time ratio, stride length, average print area, and stance pressure. The gap in these dynamic and static patterns of mice between non-operated and operated groups gradually decreased over healing time, indicating successful surgeries in the murine models. As compared to the HMP and the slit3-AB groups, the slit3 significantly increased the stance time ratio, stride length, paw-print area, and stance pressure at 1, 2, 4, and 6 weeks after surgery. As expected, the slit3-AB treatment significantly reduced the stance time ratio, stride length, paw-print area, and stance pressure relative to the HMP treatment in mice with ACL reconstruction (Figure 6B).

### 2.6. Biomechanical Testing of FTGTC

**Figure 7:**
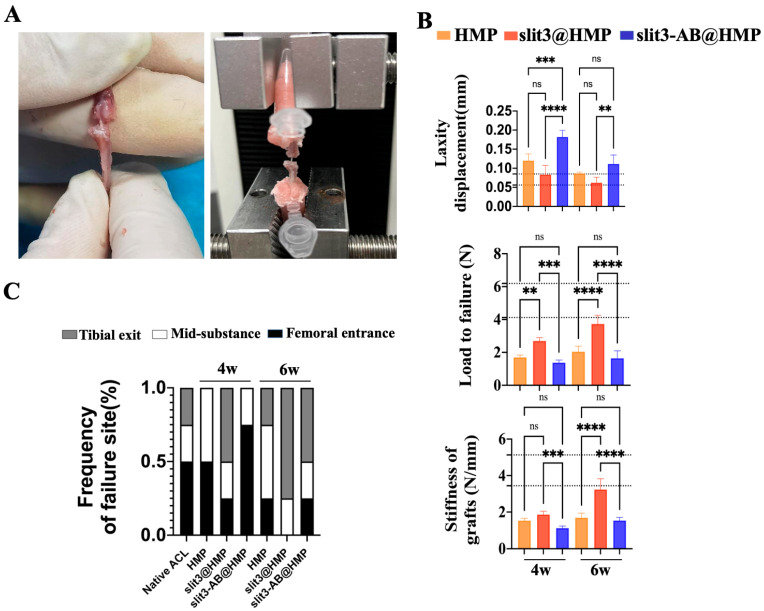
(**A**) Schematic diagram showing tensile testing of FTGTC from the HMP, slit3@HMP, and slit3-AB@HMP groups at 4 and 6 weeks after reconstruction. (**B**) Quantitative analysis of knee joint dynamic laxity displacement, maximum load to failure, and graft stiffness at 4 and 6 weeks after surgery in HMP, slit3@HMP, and slit3-AB@HMP groups at 4 and 6 weeks after reconstruction. (n = 6). The black dotted line covers the reference data range from the native knee joints in mice. ** *p* < 0.01, *** *p* < 0.001, **** *p* < 0.0001, ns: not significant. (**C**) The frequency of graft failure site during tensile testing in all the three groups at 4 weeks and 6 weeks after surgery.

After gait analysis, the tendon-graft–bone-tunnel bonding strength was then quantitatively measured by tensile tests (Figure 7A). Poor healing may cause gradual pullout of the tendon graft from bone tunnels, ultimately leading to knee joint laxity, so the dynamic knee joint laxity of mice was tested and compared. Obviously, the slit3 treatment profoundly favored the decrease in knee joint laxity displacement (0.088 ± 0.022 mm vs. 0.116 ± 0.016 mm) while the slit3-AB treatment significantly increased knee joint laxity displacement relative to the HMP group (0.169 ± 0.019 mm vs. 0.116 ± 0.016 mm, *p* < 0.001) at 4 weeks post-operation. At 6 weeks, there was no significant difference in the laxity displacement between the HMP group and the slit3-AB group (0.085 ± 0.003 mm vs. 0.112 ± 0.024 mm), but the laxity displacement in the slit3 group was still significantly lower than that in the slit3-AB group (0.063 ± 0.014 mm vs. 0.112 ± 0.024 mm, *p* < 0.01). Although there was no significant difference in laxity displacement between the HMP and the slit3 treatment groups, the slit3 treatment significantly improved the maximum load to failure relative to the HMP group at 4 weeks (2.495 ± 0.783 N vs. 1.668 ± 0.33 N, *p* < 0.01) and 6 weeks (3.845 ± 0.532 N vs. 1.973 ± 0.425 N, *p* < 0.0001) after operation (Figure 7B). Meanwhile, as compared to the HMP group, the slit3-AB remarkably reduced the load to failure. Then, the graft stiffness was calculated according to the load-displacement curves. Apparently, the slit3 treatment dramatically increased the graft stiffness while the slit3-AB decreased the graft stiffness relative to the HMP treatment at 4 weeks post-operation. At 6 weeks, there was a significant increase in the graft stiffness in the slit3-treated group when compared to the HMP group (3.23 ± 0.45 N/mm vs. 1.68 ± 0.27 N/mm, *p* < 0.0001). Encouragingly, both laxity and tensile testing showed that the mice with slit3 treatment gradually restored their pre-injury biomechanical performance at 6 weeks after surgery. Afterwards, the failure mode was recorded. Although impaired tendon–bone healing may easily lead to the graft being pulled out from the bone tunnel during the tensile testing, the insufficient graft integration would also cause knee joint laxity, resulting in degeneration of the graft mid-substance and thereby contributing to deterioration in the mechanical strength. Therefore, the failure occurrence was found at the tibial exit, mid-substance, and the femoral entrance in FTGTC during tensile testing in all the three groups after surgery (Figure 7C).

## 3. Materials and Methods

All the operations were performed with the approval by the local Animal Care and Use committee (SYSU-IACUC-2022-001534). The detailed information is described as follows.

### 3.1. Study Design

Eighty-seven 10-week-old male C57BL/C mice were purchased and housed in a specific-pathogen-free animal facility. They were divided into 3 groups for ACL reconstruction, which were treated with hydrogel microparticles (HMP) alone, slit3 loaded HMP (slit3@HMP), and slit3 neutralizing antibody (slit3-AB@HMP), respectively (n = 27 for each group). After surgery, the mice in the above three groups and the sham group were used for gait analysis at 1, 2, 4, and 6 weeks (n = 6/group/time point), general histological and immunofluorescence analyses at 2, 4, and 6 weeks post-operation (n = 5/group/time point), micro-CT scanning and assessment at 2, 4, and 6 weeks after surgery (n = 5/group/time point), and biomechanical testing for knee joint laxity displacement and load to failure at 4 and 6 weeks post-surgery (n = 6/group/time point). The flowchart of the experiment are shown in Figure 1.

### 3.2. Preparation of HMPs

To generate droplets, a microfluidic water-in-oil segmentation system was utilized. In a typical experimental configuration, the water phase consisted of an aqueous dispersion containing 1% (*w*/*v*) thiolated sodium alginate (SH-SA), 3.33% (*w*/*v*) vinyl-terminated hyperbranched (polyethylene glycol) diacrylate (HB-PEGDA), and 3.52 × 10^9^ infectious units per milliliter (IFU/mL) of adenovirus. The oil phase, on the other hand, consisted of 3M™ Fluorinert™ FC-40 supplemented with 2 wt% Pico-SurfTM. The resulting droplets were collected in an Eppendorf tube and subsequently transferred to a drying oven, where the HMPs were cured at a temperature of 37 °C for a minimum of 1 h. To complete the process, the HMPs underwent purification by introducing Novec 7500 oil containing 20% v/v 1H, 1H, 2H, 2H-perfluoro-1-octanol (PFO) to eliminate any remaining surfactants. Following this, the HMPs were allowed to swell and equilibrate with buffer for a minimum of 2 h at 37 °C prior to their utilization. Fully swollen and equilibrated building block HMPs were pelleted at 8000 rpm for five minutes, and the excess buffer was removed to give the HMPs. All the catalog and company details of the materials used in this study are shown in Table S1.

### 3.3. Characterization of HMPs

The chemical structure and composition of the polymers for preparing HMPs were verified by ^1^H Nuclear Magnetic Resonance Spectroscopy (NMR) (HAAKE RheoStress 1, Thermo Scientific, Massachusetts, USA). Particle size of HMPs was visualized using the ICX41 (SOPTOP ICX41 Sunny Optical Technology (Group) Co., Ltd., Ningbo, China) inverted optical microscope and a scanning electron microscope (SEM, S-4800 field, Hitachi, Tokyo, Japan). The rheological behaviors of the resulting HMPs was tested at 25 °C using a rheometer (HAAKE RheoStress 1, Thermo Scientific, Massachusetts, USA) with a stainless steel parallel plate rotor with a diameter of 8 mm. A dynamic oscillatory strain amplitude sweep measurement was performed by varying the strain percentage from 0.01% to 1000% at a frequency of 1 Hz. Additionally, oscillatory frequency sweeps were conducted, ranging from 0.1 to 100 rad/s at a fixed strain amplitude of 1%. Furthermore, strain-cycle experiments were carried out to assess the recovery properties of the HMPs. These experiments involved subjecting the samples to cycles of high strain (γ = 1000%) followed by low-amplitude strain (γ = 0.1%) until the monitoring resumed, allowing for the investigation of the hydrogels’ recovery behavior after failure at high strain. Moreover, flow curves were obtained through strain-rate-controlled measurements, covering a range of shear rates from 10^−2^ to 10^2^ s^−1^. These experiments provided valuable insights into the viscoelastic and flow properties of the HMPs.

### 3.4. In Vitro Cytotoxicity Assessment and Stability Testing of slit3 Loaded in HMPs

Human umbilical vein endothelial cells (HUVECs) were cultured in Minimum Essential Medium α (MEM α, Gibco, 12571071,Carlsbad, CA, USA) supplemented with 10% fetal bovine serum (FBS, Gibco,10099141, Carlsbad, CA, USA) and 1% penicillin–streptomycin at 37 °C in a humidified atmosphere containing 5% CO_2_ in an incubator (Esco Micro Pte. Ltd., CCL-170B-8, Singapore). HUVECs were seeded in the wells of 96-well culture plates at a density of 1.0 × 104 cells per well and cultured overnight for adhesion. The HMPs loaded with a series of ascending concentrations of slit3 were then added into wells for direct co-culture with HUVECs. After 48 h, the medium was removed and then replaced with 110 μL fresh culture medium containing 10 μL cell counting kit-8 (CCK8) reagents prior to incubation in a dark environment for 4 h at 37 °C. Afterwards, 80 μL CCK8-containing medium was pipetted from each well and transferred into a new 96-well plate. The absorbance at the wavelength of 450 nm was measured on a microplate reader (ALLSHENG, AMR-100, Hangzhou, China).

In addition, the cytocompatibility of slit3@HMP was assessed by Live/Dead staining assay. The HUVECs were seeded in a 48-well plate at a density of 10^4^ cells per well and cultured overnight prior to the addition of different doses of slit3@HMP solution. After 24 h, the viability of cells was analyzed using the Live/Dead assay kit (Solarbio, CA1630, Beijing, China). The images were taken using a fluorescent microscope (MSHOT, MF52-M, Guangzhou, China). Therefore, the slit3@HMP were placed into the dialysis bag (JielePu, MD10-100000, Changsha, China) for measurement of the remained slit3 at different time points by the Elisa kit (Cloud-Clone, SED353M, Wuhan, China). The slit3 alone was set as the control group.

### 3.5. Surgical Procedure

**Figure 8:**
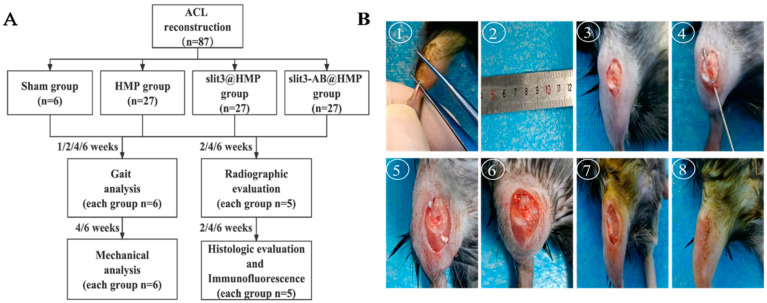
(**A**) The flowchart of the experimental design and (**B**) surgical procedures showing ACL reconstruction performed in mice. ①: isolation of tendon graft from gastrocnemius tendon; ②: fixation of two ends of the tendon graft with sutures; ③: exposure of the knee joint; ④: creation of bone tunnel and injection of HMPs; ⑤: placement of the tendon graft; ⑥: fixation of the tendon graft at the femoral exit and the tibial entrance; ⑦: relocation of knee joint; and ⑧: closure of the wound.

The mice were anesthetized through intraperitoneal injection of 1% sodium pentobarbital and 10% chloral hydrate. After anesthesia, a 5–7 mm incision was made in the back of the calf to expose the Achilles tendon. Then, an 8-0 suture (R831, Jinhuan, China) was used to fix the two ends of the gastrocnemius tendon prior to isolation from the Achilles tendon. Afterwards, an incision was made adjacent to the patellar tendon to allow the patellar bone dislocation to facilitate the creation of the bone tunnel of 0.5 mm in diameter using a bone drill along the footprints of original ACL through transtibial technique. A certain concentration of HMP (2 × 10^4^ HMPs in 1 mL PBS) was manually mixed with human recombinant slit3 or slit3-AB protein (100 μg/mL in HMP-PBS solution) for 1 h on ice for surgical use [19]. After that, 10 μL HMP, slit3@HMP, or slit3-AB@HMP was injected into the pre-drilled bone tunnels prior to the placement of the isolated tendon graft. Both of the graft ends were then sutured tightly with surrounding soft tissues around tibial entrance and femoral exit. Of note, initial graft tensioning is important for tendon–bone healing according to both animal [20] and clinical [21] studies. In our study, a constant force of approximately 0.1 N was applied to keep the graft tension during the surgery. Finally, the patellar bone was relocated to facilitate a layer-by-layer closure of incisions with absorbable sutures (Ethicon 8-0, New Brunswick, NJ, USA). The surgical procedures and experimental design were showed in Figure 8.

Analgesics including meloxicam and buprenorphine (0.1 mg per kg body weight, twice daily for 3 days) were injected for pain relief. We maintained all animals in the Animal Facility of the Sun Yat-Sen University. The experimental protocol was reviewed and approved by the Institutional Animal Care and Use Committee of Sun Yat-Sen University, Guangzhou, China.

### 3.6. Tissue Collection

For those samples for radiographic assessment, histological and immunofluorescence analyses, the mice were anesthetized with sodium pentobarbital and then fixed with a mixed solution composed of 4% paraformaldehyde and 0.1% sodium heparin (MACKLIN, 9041-08-01, Shanghai, China) through transcardiac perfusion. Afterwards, the femur–tendon graft–tibia complexes (FTGTC) were harvested and then fixed in 4% paraformaldehyde for 48 h prior to decalcification and tissue embedding.

For those samples for biomechanical testing, the mice were euthanized with overdose of anesthetics and then FTGTC was wrapped with saline gauze and stored at −80 °C.

### 3.7. Radiographic Evaluation of Peri-Tunnel Bone Tissue by Micro-Computed Tomography (Micro-CT)

The knee joint of mice was fixed in the scanning tube and scanned with the voltage, current, integration time, and isotropic voxel size set as 70 kV, 114 μA, 230 ms, and 9 μm, respectively (SCANCO μCT100, Bassersdorf, Switzerland). The bone volume/total volume fraction (BV/TV), bone mineral density (BMD), number of trabecular bone (Tb.N), trabecular thickness (Tb.Th) and trabecular separation (Tb.Sp) of the peri-tunnel bone tissue covered by a circle 0.5 mm in diameter in bone tunnels in the tibia were measured. Five samples in each group at each time point were used for statistical analysis.

### 3.8. Histological Evaluation

After micro-CT scanning, the FTGTC were decalcified with 10% ethylenediaminetetraacetic acid (EDTA). After decalcification, the samples were dehydrated serially in ascending concentrations of sucrose solution ranging from 10% to 30% for 48 h. The dehydrated samples were then embedded in optimum cutting temperature compound (OCT) for sections (10 μm) along the longitudinal direction of bone tunnels. Hematoxylin and Eosin (H&E) staining was performed to identify and measure the fibrous scar tissue, which is a kind of irregularly arranged fibrous tissue consisting of numerous spindle-shaped cells [22], at the TBI. In addition, the bony ingrowth at the TBI was assessed by Safranin O/Fast Green (SO/FG) staining. The images were captured and then analyzed by two blinded researchers using ImageJ (NIH, Bethesda, MD, USA). The microscopic images were acquired using a microscope (BDS400, CQOPTEC, Chongqing, China).

### 3.9. Immunofluorescence Staining

The tissue sections were transferred directly into antigen retrieval solution (sodium citrate, pH 6.0) and then blocked with a mixture of 3% goat serum, 1% bovine serum albumin (BSA), and 0.1% Triton X-100 in phosphate-buffered saline (PBS) for 1 h at room temperature. Subsequently, the sections (20 μm) were probed overnight with the primary antibodies at 4 °C and then incubated with the secondary antibodies for 1 h at room temperature. The cell nuclei were counterstained with DAPI and the images were recorded using confocal laser scanning microscope (FV3000, Olympus, Tokyo, Japan). The following primary antibodies were used: Endomucin (sc-65495, SANTA CRUZ, Dallas, TX, USA) and CD31 (ab28364, Abcam, Cambridge, UK). The secondary antibodies used included anti-rat Alexa Fluor 488 (4416, Cell Signaling, Boston, MA, USA) and anti-rabbit Alexa Fluor 649 (BS10034, Bioworld, Dallas, TX, USA).

### 3.10. Biomechanical Testing

The frozen FTGTC were thawed at the room temperature for measurement of dynamic laxity and maximum load to failure. All the soft tissue but the tendon grafts were carefully removed. Then, the two ends of FTGTC were fixed by denture base resins in a 0.5 mL centrifuge tube prior to fixation in jigs. After that, the assessment of dynamic joint laxity was performed with the knee in full extension at a speed of 2 mm/min in a cyclic loading model between 0.1 N and 1 N on a mechanical testing machine (Zhiqu, ZQ-990LB, Dongguan, China). After 10 cycles, the resultant displacement was recorded. After the laxity test, the tensile test for the maximal load to failure was conducted at a speed of 2 mm/min until an abrupt drop in loading curve. The maximal load and the model of failure were recorded. The stiffness was calculated at linear region of the load-displacement curve.

### 3.11. Gait Analysis

The gait performance of mice in the sham group, the HMP-treated group, the slit3@HMP-treated group, and the slit3-AB@HMP-treated group at 1, 2, 4, and 6 weeks post-surgery were analyzed. The gait behavior of the mice without surgery was also recorded for comparison. The mice were placed on a flat glass runway (40 × 8 cm) and allowed to walk freely. After the mice were adapted to walk, a high-speed camera was used to record the animal footprints under the runway to collect data on Stance time ratio, Stride length, Average print area, and Stance pressure by the gait analysis software Runway Scan (CleverSys, Reston, VA, USA).

### 3.12. Statistical Analysis

The data are expressed as mean ± standard deviation (mean ± SD). SPSS 22.0 software (IBM Corp., Armonk, NY, USA) was used for data analysis, and Prism Version 8 (GraphPad Software) was applied for image drawing. The independent *t*-test was used for comparison of data between two groups, and one-way analysis of variance (ANOVA) with Bonferroni post hoc test was used for multiple comparisons in over two groups. *p* < 0.05 was considered statistically significant and the statistical significance was declared as (*) at *p* < 0.05, (**) at *p* < 0.01, (***) at *p* < 0.001, and (****) at *p* < 0.0001. Animal surgeries and data analysis were blindly performed by different investigators.

## 4. Discussion

This is the first study to investigate the effects of slit3 treatment on tendon–bone healing through modulation of type H vessel formation at the TBI in mice models with ACL reconstruction. Increased CD31^hi^Emcn^hi^ endothelium was observed at the TBI after slit3 treatment while the opposite result was detected when the slit3-AB was applied. Concomitantly, the slit3 treatment favored the bony ingrowth towards the TBI, contributing to improved gait performance and load to failure with less laxity displacement. The results indicated that the local injection of slit3 may be a promising approach to accelerate and enhance the graft osteointegration into bone tunnels in patients with ACL reconstruction.

The TBI structure is highly complex and heterogeneous, making it challenging for the graft osteointegration into bone tunnels. Osteogenesis is strictly connected to angiogenesis and jointly contributes to new bone formation, so it is critical to understand the crosstalk between endothelium and osteoprogenitors. In addition to promoted osseous growth into the TBI, less fibrous scar tissue is expected for satisfactory graft healing. Therefore, some typical angiogenic factors, such as VEGF and PDGF-BB, may not be recommended for modulation of angiogenesis at the TBI due to their potential involvement in pro-inflammatory responses [23]. Most recently, osteoblast- and osteoclast-derived slit3 has been found to be a novel angiogenic factor favoring type H vessel formation via activation of slit3/Roundabout guidance receptor 1 (Robo1)-dependent signaling in the endothelium progenitor cells [13]. More importantly, the enhancement of type H vessel formation facilitates the osteogenic differentiation of surrounding enriched osteoprogenitors. Simultaneously, the slit3 secreted by the increased osteoblasts further favors type H vessel formation, thereby contributing to the onset of coupling effects between angiogenesis and osteogenesis.

Generally, type H vessels have been identified in specific locations, mainly in metaphysis near the growth plate and both the periosteum and endosteum of the diaphysis [24]. In terms of ACL reconstruction, the pre-drilled bone tunnels through the metaphysis around the growth plate may support type H vessel formation at the TBI. Consistent to our hypothesis, there was type H vessel formation at the TBI in the HMP group at 2 weeks after surgery. In this study, as a result of the short half-life in the physiological environment of slit3 [25], we considered to deliver slit3 through in situ injection of the slit3-loaded HMPs, aiming to improve the structural stability of slit3 for extended release. As supported by the release behavior in Figure 2C, the slit3 loaded in HMPs exhibited much better stability relative to the slit3 alone in the simulated solution. Encouragingly, the slit3 treatment remarkably increased the type H vessel formation while the slit3-AB decreased the CD31^hi^Emcn^hi^ signal. Apparently, the modulation of type H vessel formation at the TBI caused changes in knee joint functions. The histological analysis indicated that the promoted type H vessel at the TBI favored bony ingrowth towards the tendon graft. Importantly, the promoted angiogenesis did not induce more fibrous scar tissue formation. The enhanced osseous ingrowth at the TBI was also accompanied with increased peri-tunnel bone mass and decreased tunnel size, indicating that the local injection of slit3 affected bone remodeling at both the TBI and the peri-tunnel tissue. The aim of ACL reconstruction is to restore the pre-injury activity level as much as possible in an accelerated pattern. The gait analysis was then recorded and compared. Consistent to the histological and radiographic findings, the slit3 treatment significantly improved the gait performance of mice relative to the HMP group. Although a series of cytokines exhibit angiogenesis or osteogenesis effects, their in situ injection may still not be recommended in ACL reconstruction due to their pro-inflammatory properties (e.g., VEGF, PDGF-BB, and calcitonin gene-related peptide, etc.) [26], which would cause pain, impairing gait performance. Therefore, slit3 may be considered as a potential angiogenic factor suitable for in situ injection into bone tunnels without causing extra pain and inflammatory responses. As expected, increased bony ingrowth into the TBI contributed to improved biomechanical performance. Although only 6 weeks were selected to monitor the graft healing in mice, the biomechanical parameters of the slit3-treated mice, including knee joint laxity displacement, the maximum load to failure, and the graft stiffness, showed values very close to the reference range in the normal mice.

There are still some limitations in this study. First, we did not monitor the in vivo release behavior and tissue distribution of slit3. In the following study, we will label slit3 with a fluorescent probe for real-time imaging. Second, a murine model was used and the findings may not be directly applicable to clinical practice. A beagle model will be considered to test the translational potential of in situ injection of slit3 loaded hydrogels in our future work. In addition, an extended period will be designed to assess the complete healing process in the beagle dog model after ACL reconstruction to address the concern with the short observation time. Third, sex is an important factor influencing tendon or bone healing [27,28], so we just considered a single-sex group in this study to investigate if the type H vessel affects the graft osteointegration towards the bone. It will be interesting to conduct the experiments in female mice to verify the findings observed in the male mice in the future. Finally, a delivery system targeting CD31^hi^Emcn^hi^ vascular endothelial cells for in situ release of slit3 may be a better strategy for the modulation of type H vessel formation at the TBI.

## 5. Conclusions

In this study, we demonstrated that the local injection of slit3 significantly accelerated and improved the osteointegration of the tendon graft into bone tunnels in mice after ACL reconstruction, evidenced by histological assessment, the peri-tunnel bone mass measurement, gait analysis, and biomechanical testing. The type H vessel, a specific subtype of capillary, may be a novel switch modulating the coupling effects between angiogenesis and osteogenesis at the TBI, so this work may provide a new therapeutic target in sports medicine.

## Data Availability

The Data Availability Statement Material mentioned in the article is available upon reasonable request.

## Supplementary Materials

The following supporting information can be downloaded at: https://www.mdpi.com/article/10.3390/ijms241713638/s1.
