# Supplementary material for: Slit Guidance Ligand 3 (SLIT3) Loaded in Hydrogel Microparticles Enhances the Tendon-Bone Healing through Promotion of Type-H Vessel Formation: An Experimental Study in Mice
Source: Int J Mol Sci. 2023 Sep 4;24(17):13638. doi: 10.3390/ijms241713638 (PMC10488208; doi:10.3390/ijms241713638)
Supplement: Supplementary file 1 [file ijms-24-13638-s001.zip › ijms-2482416-supplementary.pdf]

Table S1 Detailed information of the used materials in this study

Names of Materials	*Number*	*Company*
Sodium alginate(SA)	*CAS No. 9005-38-3*	Aladdin Shanghai Reagent Co.,Ltd (Shanghai, China)
dithiothreitol (DTT)	*CAS No. 3483-12-3*	Aladdin Shanghai Reagent Co.,Ltd (Shanghai, China)
3, 3′-Dithiobis (propanoic dihydrazide) (DTP)	*CAS No. 50906-77-9*	Frontier Scientific Co., Ltd, USA
1-Ethyl-3-(3-dimethylaminopropyl) carbodiimide hydrochloride (EDCI)	*CAS No. 25952-53-8*	Damas-beta Co., Ltd (Shanghai, China)
2-Morpholinoethanesulphonic acid(MES)	*CAS No. 4432-31-9*	Aladdin Shanghai Reagent Co.,Ltd (Shanghai, China)
Poly (ethylene glycol) diacrylate (PEGDA) (average Mn 575)	*CAS No. 26570-48-9*	Sigma-Aldrich Co., Ltd, Shanghai, China
2,2′-Azobis(2-methylpropionitrile) (AIBN)	*CAS No. 78-67-1*	Aladdin Shanghai Reagent Co.,Ltd (Shanghai, China)
Tetraethylthiuram disulfide (DS)	*CAS No. 97-77-8*	Aladdin Shanghai Reagent Co.,Ltd (Shanghai, China)
Fluorinert™ FC-40	*CAS No. 86508-42-1*	3M
1H, 1H, 2H, 2H-perfluoro-1-octanol (PFO)	*CAS No. 647-42-7*	Bide Pharm Co., Ltd (Shanghai, China)
Novec™ 7500	*CAS No. 297730-93-9*	3M
Minimum Essential Medium α (MEM α)	*12571071*	Gibco, USA
fetal bovine serum (FBS)	*10099141*	Gibco, USA
counting kit-8(CCK8)	*HY-K0301*	MCE, USA
Live/Dead assay kit	*CA1630*	Solarbio
Slit3 Elisa kit	*SED353M*	Cloud-Clone, China
4% paraformaldehyde	*BL539A*	Biosharp, USA
8-0 suture	*R831*	Jinhuan, China
sodium heparin	*9041-08-01*	*MACKLIN, China*
ethylenediaminetetraacetic acid (EDTA)	*6381-92-6*	*MACKLIN, China*
optimum cutting temperature compound (OCT)	*4583*	TissueTek, USA
Hematoxylin and Eosin dye	*PH0512*	Phygene, China
Safranin O/Fast Green	*RBG1036-200-2*	Roles-Bio,
antigen retrieval solution	*BL618A*	Biosharp, USA
bovine serum albumin (BSA)	*9048-46-8*	Biofroxx, German
Triton X-100	*9002-93-1*	Sigma, USA
DAPI	*S2110*	Solarbio, China
Slit3 Elisa kit	*SED353M*	Cloud-Clone, China
4% paraformaldehyde	*BL539A*	Biosharp, USA
8-0 suture	*R831*	Jinhuan, China
Endomucin antibody	*sc-65495*	SANTA CRUZ, USA
CD31 antibody	*ab28364*	Abcam, USA

anti-rat Alexa Fluor 488	*4416*	Cell signaling, USA
anti-rabbit Alexa Fluor 649	*BS10034*	Bioworld, USA
Slit3	*767804*	Biolegend, USA
